# Oxidative Stress in Rheumatoid Arthritis: What the Future Might Hold regarding Novel Biomarkers and Add-On Therapies

**DOI:** 10.1155/2019/7536805

**Published:** 2019-12-14

**Authors:** Lucas José Sá da Fonseca, Valéria Nunes-Souza, Marília Oliveira Fonseca Goulart, Luiza Antas Rabelo

**Affiliations:** ^1^Laboratório de Reatividade Cardiovascular, Setor de Fisiologia, Núcleo de Síndrome Metabólica, Instituto de Ciências Biológicas e da Saúde, Universidade Federal de Alagoas, Maceió, Alagoas, Brazil; ^2^Departamento de Fisiologia e Farmacologia, Centro de Biociências (CB), Universidade Federal de Pernambuco (UFPE), Recife, Pernambuco, Brazil; ^3^Max-Delbrück-Center for Molecular Medicine, Berlin, Germany; ^4^Instituto Nacional de Ciência e Tecnologia em NanoBiofarmacêutica (N-BIOFAR), Belo Horizonte, Brazil; ^5^Instituto de Química e Biotecnologia (IQB), Universidade Federal de Alagoas, Maceió, Alagoas, Brazil

## Abstract

Numerous rheumatologic autoimmune diseases, among which rheumatoid arthritis, are chronic inflammatory diseases capable of inducing multiple cumulative articular and extra-articular damage, if not properly treated. Nevertheless, benign conditions may, similarly, exhibit arthritis as their major clinical finding, but with short-term duration instead, and evolve to spontaneous resolution in a few days to weeks, without permanent articular damage. Such distinction—*self-limited arthritis with no need of immunosuppressive treatment or chronic arthritis at early stages?*—represents one of the greatest challenges in clinical practice, once many metabolic, endocrine, neoplastic, granulomatous, infectious diseases and other autoimmune conditions may mimic rheumatoid arthritis. Indeed, the diagnosis of rheumatoid arthritis at early stages is a crucial step to a more effective mitigation of the disease-related damage. As a prototype of chronic inflammatory autoimmune disease, rheumatoid arthritis has been linked to oxidative stress, a condition in which the pool of reactive oxygen species increases over time, either by their augmented production, the reduction in antioxidant defenses, or the combination of both, ultimately implying compromise in the redox signaling. The exact mechanisms through which oxidative stress may contribute to the initiation and perpetuation of local (in the articular milieu) and systemic inflammation in rheumatoid arthritis, particularly at early stages, still remain to be determined. Furthermore, the role of antioxidants as therapeutic adjuvants in the control of disease activity seems to be overlooked, as a little number of short studies addressing this issue is currently found. Thus, the present review focuses on the binomial *rheumatoid arthritis-oxidative stress*, bringing insights into their pathophysiological relationships, as well as the implications of potential diagnostic oxidative stress biomarkers and therapeutic interventions directed to the oxidative status in patients with rheumatoid arthritis.

## 1. Introduction

Rheumatoid arthritis (RA), a systemic autoimmune disease with a characteristic pattern of joint destruction, is far long recognized as a clinical condition associated with potential compromise through diverse fronts, with organ damage not only restricted to the musculoskeletal system [1–5]. With regard to the disease course along time, great efforts have been made in recent years in order to differentiate early stages of the disease from those not associated to autoimmunity [1, 6, 7]. The importance of such distinction came to light by the observation that the pathophysiological mechanisms involved in the initiation and establishment of RA change as the time goes by, with some inflammatory mediators predominantly present at the early clinical disease and not markedly observed in advanced stages of joint damage [8–10]. The comprehension of this mutable pattern of effector cytokines in RA does have potential therapeutic implications, drawing the concept of *window of opportunity*, that is, the utmost opportune moment to therapeutically intervene [4, 8], in order to block local and systemic inflammatory cascades, aiming to have better control of disease activity and, consequently, better clinical outcomes [3, 6, 11].

Considering the well-recognized connection between oxidative stress and chronic inflammation [12–15], the former also known as redox imbalance, described as a condition in which the pool of reactive oxygen species (ROS) increases over time, either by their augmented production, the reduction in antioxidant defenses, and/or the combination of both [14, 16], ultimately leading to impaired redox signaling and/or compromise in the control of molecular damage [17], it becomes easy to admit the potential cross-talk between oxidative stress and RA, as such autoimmune disease characteristically represents an entity of chronic systemic inflammation [5, 18, 19]. Despite this almost *self-explained* interaction, very few studies were devoted to the comprehension of the in-depth mechanisms through which redox imbalance may contribute to the establishment of the proinflammatory milieu observed in RA and *vice versa*, with fewer studies focusing on the study of oxidative biomarkers in such autoimmune arthritis [12, 19, 20].

**Figure 1:**
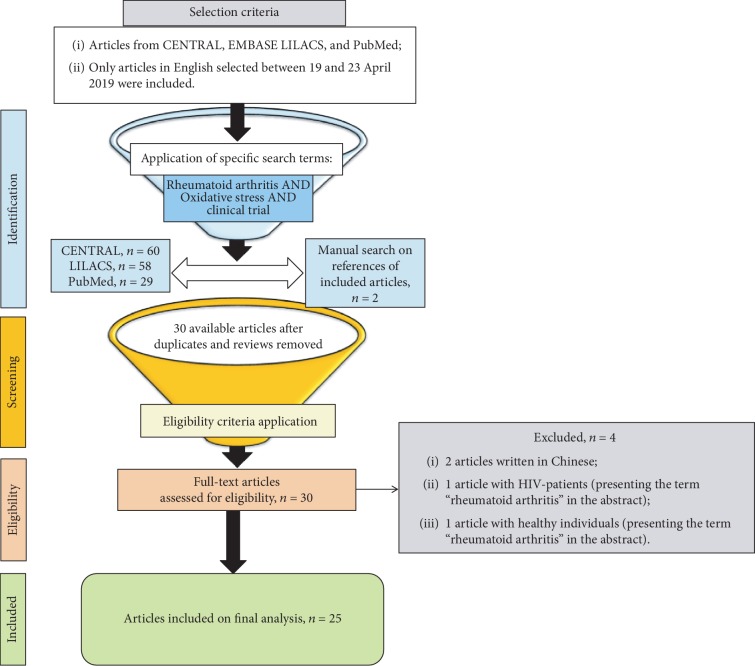
Workflow through the different phases of the review.

In the context of differential diagnosis, however, many metabolic, endocrine, neoplastic, granulomatous, infectious diseases and other autoimmune conditions may mimic RA [3, 8, 10], the reason why one might ask: *how to say for sure it is RA what is being talked about if, at early stages, too many mimickers may challenge us?* The hope for answering this question seems to rest on the identification of biomarkers and/or the development of properly validated clinical models capable of predicting the risk of progression to chronic arthritis in patients at early stages of a yet undifferentiated arthritis [1, 6, 8, 21]. Thus, the present review focuses on the binomial *rheumatoid arthritis-oxidative stress*, bringing insights into their pathophysiological relationships, as well as the implications of potential diagnostic oxidative stress biomarkers and therapeutic interventions directed to the oxidative status in patients with RA. The search for clinical trials based on antioxidant complementary treatments in RA used the combination of descriptors “Oxidative Stress AND Rheumatoid Arthritis AND Clinical Trial” in three different databases, namely, CENTRAL, EMBASE, and PubMed.  Figure 1 summarizes the sequence of selection till the final analysis.

## 2. The Binomial Rheumatoid Arthritis-Oxidative Stress

### 2.1. Early Disease in Rheumatoid Arthritis: Earlier Than First Thought

RA, a chronic inflammatory disease affecting mainly the synovial tissue [4, 8, 9] with persistent synovitis [22–24] and a destructive articular pattern [25], occupies the top position in the list of systemic autoimmune diseases [4].

**Figure 2:**
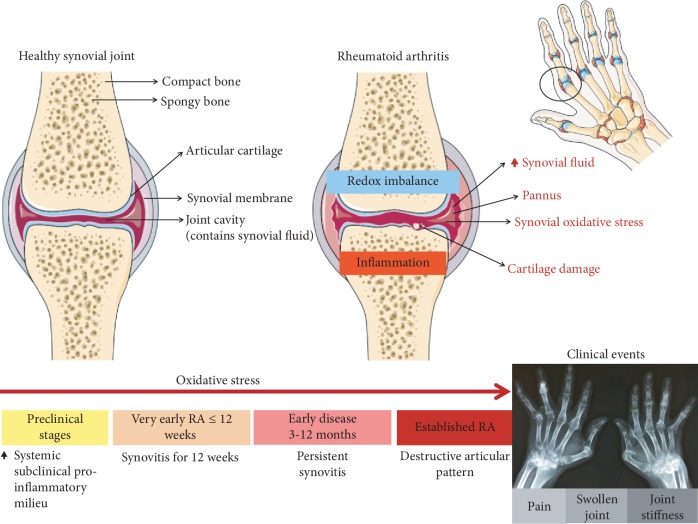
Marching on towards joint destruction: the timeline of rheumatoid arthritis. Years or even decades before clinical disease, rheumatoid arthritis individuals may already exhibit elevated levels of circulating autoantibodies in a systemic subclinical proinflammatory milieu. As the intra-articular oxidative stress and local inflammatory mediators, either cellular or soluble ones, take their places in disease progression, the clinical manifestations become apparent, manifested mainly by joint pain and swelling, with consequent inflammatory morning stiffness. This occurrence characterizes the prototype of inflammatory arthritis, which if not properly treated, preferentially at early stages, might evolve to permanent articular damage, with prominent joint destruction and disability. RA: rheumatoid arthritis.

The concepts of disease duration for RA are not consensual in the literature [7], but in general, *early disease* has been referred to as that with duration of at least 3 months [1, 7] and less than 12 months [25], once disease duration superior to one year characterizes what has been called *established RA* [4]. Also, in an attempt to reinforce the importance of prompt diagnosis and establishment of directed pharmacological treatment as soon as possible, some studies go further in such conceptual aspect and define what is called *very early arthritis* [7, 8], a term that would be reserved to clinical presentations with no longer than 12 weeks of duration [7, 26]. Such cut-off point was defined by the observation that at 3 months, the disease pattern is already established: the presence of synovitis for 12 weeks increases the likelihood of evolving to chronic inflammatory joint disease, so that shorter durations are associated with better prognosis [7, 8] (Figure 2). In line with the efforts to more adequately understand the progression of the immunopathogenic pathways along the disease course, a novel classification by stages has been recently suggested: *triggering phase*, with individuals genetically predisposed to RA exposed to diverse environmental triggers; *maturation phase*, in individuals without synovitis but positive for anticitrullinated protein antibodies (ACPA); *targeting phase*, in individuals seropositive for ACPA with arthralgia; and *fulminant disease*, characterized by established RA [18].

Approximately one percent of the general population carries the disease [23, 27, 28], and its concept of being a systemic condition highlights the fact that the burden of RA, when it comes to body damage, is not only restricted to joints [4, 5, 24, 25]. Indeed, the systemic inflammatory milieu observed in RA patients leads them to increased cardiovascular risk [4, 5, 25, 29], besides potential damage to different organs, including, but not only restricted to, the eyes [30], lungs [24, 31], heart [30, 32], small vessels [4], and skin [30], ultimately implying higher probability of premature death [21, 33, 34].

Considering that the articular compromise in RA may last for decades, the idea that the early stages of the disease would stand for periods as long as many months to years could sound as a plausible concept [10], allowing the application of the *watch and wait policy*, that is, waiting for initiating pharmacological treatment only when no doubt about the diagnosis of RA existed [8]. This conduct, however, does not seem to be adequate, once recent cohort studies and others have shown that the early stages of the disease are actually earlier than first thought [6, 7, 9, 23, 24, 26], with the need of prompt treatment with disease-modifying antirheumatic drugs (DMARDs) as soon as the diagnosis is made, considering the predictors of high risk for evolving with chronic arthritis [6, 30].

The aforementioned definitions in the timeline of RA have been recently emphasized because of the recognition, in the last two decades, that the immunopathogenic pathways in the initial course of the disease differ from those observed in advanced stages [9, 21–23, 25, 26]. And what is more, in preclinical stages of RA, a systemic subclinical proinflammatory milieu is already recognized [9, 25], with the identification of circulating autoantibodies until 18 years before the clinical manifestations of the disease, reinforcing the idea that the immune dysregulation in RA patients is triggered even before the disease is clinically manifested [9, 24]. Thus, the clinically observed RA may not simply represent a disease, but a spectrum of presentation of a syndrome [30] with multiple interconnected pathogenic pathways, which takes its place silently before it could be even dreamed of [25].

### 2.2. Back in Time: What History Tells Us about the Binomial *Oxidative Stress-Rheumatoid Arthritis*

Pathophysiologically, RA is still not fully understood [20, 28, 35–37]. The first references mentioning the term “rheumatoid arthritis” found in the PubMed database come from 1876, represented by two citations [31, 38]. Nevertheless, the journey for the comprehension towards the linkage between oxidative stress and RA is much younger, dating back to approximately 30 years. In an interesting study, Koster and colleagues demonstrated lower concentrations of serum sulphydryl groups in RA patients compared to healthy controls. Considering that sulphydryl groups may act as scavengers of peroxides, such finding, at that time, already pointed to concrete evidence of oxidative stress in RA patients [39].

Ever since, different studies have focused on the oxidative status as a potential contributor in the initiation and establishment of a proinflammatory milieu in individuals with RA. Currently, literature undoubtedly points to the oxidative stress signature in the pathogenesis of RA [12, 13, 15, 19, 20, 40]. Oxidative stress is described as a deleterious condition characterized by a negative balance in the pool of oxidative molecules, favoring the predominance of prooxidants [14, 16], that is, ROS and reactive nitrogen species (RNS). These species, mainly represented by nitric oxide (^·^NO) [41–44], superoxide anion radical (^·^O_2_^−^) [45], peroxynitrite anion (ONOO^−^) [46, 47], hydroxyl (^·^OH) [48], lipoperoxide (LOO^·^) [14, 16], hydrogen peroxide (H_2_O_2_) [49, 50], and hypochlorous acid (HClO) [51], are highly reactive molecules generated during physiological cellular processes, as well as under several pathological conditions [9, 12, 14, 20, 47, 48, 51, 52].

Besides the potentially increased amounts of ROS/RNS in situations of oxidative stress, antioxidants also act as regulatory players, as they are substances or compounds capable of scavenging ROS/RNS and, thus, inhibiting the oxidation process in cells [16, 52]. Two different classes of antioxidants exist, namely, the enzymatic and nonenzymatic systems. The first type is mainly represented by superoxide dismutase (SOD) [52–54], catalase (CAT) [55], glutathione peroxidase (GPx) [56], glutathione reductase (GR) [57], and thioredoxin reductase [58]. ^·^O_2_^−^ and H_2_O_2_ represent the most produced ROS [16, 56], with the former scavenged by SOD [52] and the latter by CAT [55], GPx [56], and peroxiredoxins (PRX) [59]. The nonenzymatic antioxidant system, in turn, gathers some vitamins (A, C, and E), *β*-carotene, and antioxidant minerals, such as copper, ferritin, zinc, manganese, and selenium [16, 60].

In this regard, the use of oxidative stress biomarkers as a promising additional alternative in assessing disease activity and prognosis in RA patients has evolved, being recently emphasized [4, 20]. Accordingly, a 2016 meta-analysis, by evaluating clinical trials with RA individuals to study oxidative biomarkers as adjuvants in monitoring disease progression, found a positive correlation between lipid peroxidation (assessed by the serum levels of malondialdehyde (MDA)) and disease activity (evaluated by the disease activity score DAS-28), reinforcing the assumption that oxidative stress and disease activity in RA move towards the same direction. In addition, the authors highlight the potential applicability of oxidative biomarkers not only for complementary assessing disease activity but also for prognostic purposes [20].

During the preparation of the current review article, among the 813 articles found in the PubMed database in April 2019 for the combined descriptors “Rheumatoid Arthritis AND Oxidative Stress”, in this amount included 192 review articles, only 29 clinical trials were found addressing oxidative stress as a potential source of measurable biomarkers and as a therapeutic target in RA (Figure 1). Two studies were only available as abstracts, because full texts were published in Chinese (Figure 1). Such numbers evidence the relative lack of clinical studies aiming at investigating the cross-talk between the binomial *oxidative stress-rheumatoid arthritis*, and thus, the *state of the art* points to oxidative stress as a broad field in the seek for biomarkers and new complementary therapeutic interventions with potential to be added to conventional treatment options currently available for RA.

### 2.3. Oxidative Stress and Local Inflammation: Where Destruction Journey Begins

**Figure 3:**
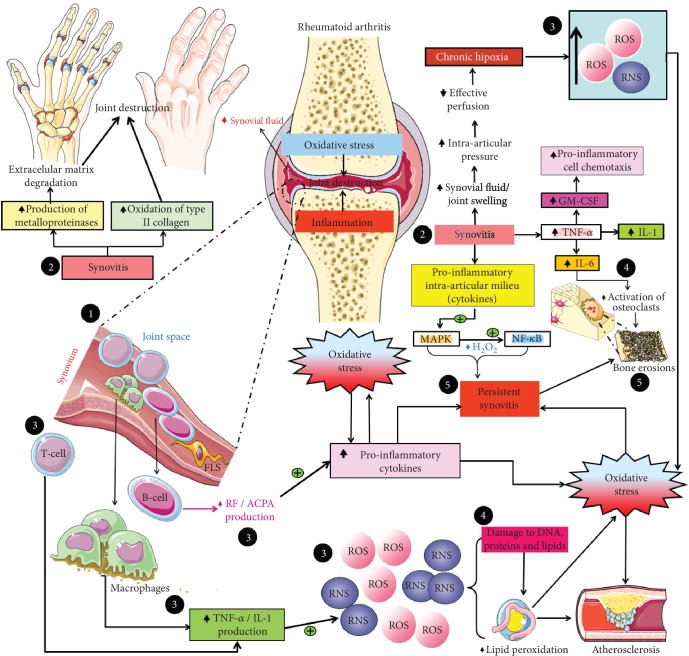
Cellular and molecular mechanisms of oxidative stress and inflammation in rheumatoid arthritis. Multidirectional interconnections are seen in the cellular and molecular mechanisms involved in the initiation and progression of articular damage in rheumatoid arthritis, so that oxidative stress may imply increased inflammation and *vice versa*, ultimately leading to a vicious cycle through which the hallmark of rheumatoid arthritis, i.e., synovitis, becomes established. (1) Arrival of inflammatory cells in the synovium; (2) establishment of synovitis; (3) soluble proinflammatory mediators produced by inflammatory cells; (4) direct effector mechanisms (cell activation, transcription of proinflammatory genes); (5) persistent synovitis and irreversible articular damage. ACPA: anticitrullinated protein antibodies; FLS: fibroblast-like synoviocyte; GM-CSF: granulocyte-macrophage colony-stimulating factor; H_2_O_2_: hydrogen peroxide; IL-1: interleukin 1; IL-6: interleukin 6; NF-*κ*B: nuclear factor-*κ*B; MAPK: mitogen-activated protein kinase; RF: rheumatoid factor; RNS: reactive nitrogen species; ROS: reactive oxygen species; TNF: tumor necrosis factor.

Oxidative stress configures a critical contributor in the initiation and maintenance of pathogenic mechanisms observed in systemic inflammatory conditions, including RA [12, 19, 29, 33, 34, 61, 62]. When it comes to physiological conditions, the production and clearance of ROS and RNS should be maintained, ideally, in a dynamic balance, once they exert pleiotropic effects on growth, differentiation, chemotaxis, and cell death [15], being also crucial in the defense mechanisms against pathogens [14]. Under pathological conditions, however, such molecules, produced at great rates by articular neutrophils, monocytes, and macrophages [63], are capable of damaging different cell structures, including DNA, carbohydrates, proteins, and lipids [13, 14, 37, 61, 64], contributing to the establishment of oxidative stress (Figure 3). In this regard, the ROS/RNS most commonly found in affected joints are represented by ^·^O_2_^−^, H_2_O_2_, ^·^OH, NO^·^, ONOO^−^, HOCl, and LOO^·^, besides the reactive compound hydrogen sulfide (H_2_S) [14, 16, 44, 48, 50].

Concerning the linkage between proinflammatory cells and oxidative stress mediators, activated macrophages and T cells in the synovium, for example, may induce the production of ROS via tumor necrosis factor (TNF) and interleukin (IL) 1 release [36], this way amplifying synovitis [37]. And what is more, a positive feedback between oxidative stress and inflammation is already recognized (Figure 3), through which both players amplify the damaging action of each other [12, 14, 65].

One of the most important pathways involved in the pathogenesis of RA, defined as a high-grade inflammation condition [4], is directly connected with oxidative stress. In this respect, proinflammatory cytokines are responsible for activating the mitogen-activated protein kinase (MAPK), which, in turn, implies the subsequent activation of nuclear factor-*κ*B (NF-*κ*B). Such molecule induces the transcription of diverse genes associated with the maintenance of inflammation [66]. Considering that ROS, mainly H_2_O_2_ [15], may activate the NF-*κ*B pathway, it becomes evident that oxidative stress is associated with the molecular signaling dysregulation observed in the early phases of RA [67]. Furthermore, NF-*κ*B may not only imply the augmented production of IL-1 and TNF-*α* but could also be activated by these proinflammatory cytokines, thus establishing a positive feedback in a self-activation process with each other [14] (Figure 3).

In this direction, RA patients with active disease, for example, present with increased levels of ROS and diminished antioxidant potential, ultimately resulting in worse oxidative status for these individuals when compared to healthy controls [13]. Consequently, a greater degree of lipid peroxidation may be found [13, 15], either in the synovial fluid or in blood samples from RA individuals [68, 69]. Accordingly, serum levels of MDA, a marker of lipid peroxidation, have been described as presenting positive correlation with proinflammatory cytokines in RA [28], with reactive oxygen metabolites (ROM) in blood samples also found to be increased in patients with RA and positively correlated with disease activity [61]. In line with these observations, lower levels of antioxidants are also found in serum and synovial fluid of RA patients [70].

When it comes to the effects of oxidative stress on specific cellular types, ROS may induce death of chondrocytes, particularly contributing to the articular damage observed in early stages of RA [15]. Furthermore, the immune dysregulation seen in autoreactive T lymphocytes may be related to their exposure to an environment of oxidative stress [4]. In addition, the proinflammatory intra-articular cascades may be amplified by the direct production of ROS by local macrophages [13] and by the local production of ACPA [4], as both rheumatoid factor (RF) and ACPA are locally produced by B cells found in the synovial tissue [25] (Figure 3).

Besides the participation of chemical mediators, physical stimuli could also contribute to the continuum of joint oxidative stress, as the increased intra-articular pressure secondary to inflammation may be responsible for chronic hypoxia, augmenting the production of ROS in joints from RA individuals [4]. Of note, not only functional cell alterations are observed in the context of oxidative stress, a fact that could be illustrated by structural extracellular changes including the oxidation of type II collagen in joints from RA patients [15], as well as by the increased production of matrix metalloproteinases [66], resulting in extracellular oxidative damage [15]. Of particular importance is the collagen oxidation described above, as it is responsible for increasing immunogenicity of extracellular matrix, contributing to amplify the loss of self-tolerance to extracellular components [14] (Figure 3).

Equally interesting, at the intracellular microenvironment, somatic mutations in p53 in fibroblast-like synoviocytes (FLS) induced by oxidative stress may contribute to synovial hyperplasia and the consequent formation of *pannus* [15], that is, the excessively proliferated synovial membrane with invasive behavior rich in CD4+/T lymphocytes [71], directly contributing to cartilage destruction and bone erosions [27, 69] and reinforcing the broad spectrum of oxidative damage in RA [13, 15].

### 2.4. Oxidative Stress and Systemic Inflammation: Far beyond the Articular Damage

Chronic inflammation, a prominent feature of RA, contributes to the increment in cardiovascular risk in its carriers [33, 34], as the risk factors classically described for cardiovascular morbimortality, namely, smoking, hypertension, diabetes mellitus, obesity, and sedentary lifestyle, do not completely explain the increased cardiovascular risk observed in RA patients [29].

Twenty years ago, the similarities between RA and the atherosclerotic disease were already described, with the observation of augmented levels of TNF, metalloproteinases, IL-6, C-reactive protein (CRP), and endothelin in both conditions. The same was true for neoangiogenesis and the expression of adhesion molecules, among which P-selectin, E-selectin, intercellular adhesion molecule 1 (ICAM-1), and vascular cell adhesion molecule (VCAM-1), strengthening the link between those vascular and articular inflammatory diseases [72]. These pathophysiological similarities point to the observation that the inflammatory mechanisms observed in RA may contribute to the establishment of endothelial dysfunction in such individuals, facilitating the understanding of the phenomenon of increased cardiovascular risk in RA patients [73].

In the context of vascular damage, endothelial dysfunction in RA patients is largely described, either in macrocirculation or in microcirculation, with reports of positive correlation between disease activity and microvascular dysfunction in early RA. Interestingly, such microvascular dysfunction may be identified even in patients with disease in remission, which evidences that, probably, there must exist other contributors but disease activity alone implying microvascular endothelial dysfunction in clinically controlled patients [4]. Furthermore, reinforcing the association between oxidative stress and inflammation in RA-related vascular dysfunction, IL-1, a proinflammatory cytokine present both at preclinical and chronic stages of RA [9], is capable of inducing oxidative stress, with consequent vascular damage in RA individuals. In fact, in such patients, improved endothelial function by the inhibition of the action of such cytokine could be observed [32].

As a systemic inflammatory disease, RA may also affects other tissues and organs through its extra-articular manifestations, besides vascular beds reported above, as exemplified by subcutaneous nodules and leg ulcerations, systemic vasculitis, pulmonary fibrosis, scleritis and episcleritis, valvular heart disease and conduction abnormalities, and cervical myelopathy, among others seen in diseased individuals [30].

## 3. Antioxidant Therapy in Rheumatoid Arthritis

Oxidative stress is a pivotal player in the aggravation of chronic inflammatory joint disease. Both experimental models and assessments in patients showed, in addition to elevated ROS and lipid peroxidation formation, a decrease in antioxidant defenses [13, 14, 60, 67]. In this sense, antioxidant therapy may possibly offer novel adjuvant/complementary treatment options aiming at better controlling disease activity [37], as many patients do not achieve remission/low disease activity with the currently available pharmacological treatment [9].

The involvement of oxidative stress in the pathogenesis of inflammatory diseases, such as RA, has been demonstrated in several studies [20, 32, 67, 69, 74]. ROS and RNS are important categories of molecules generated in living systems for cellular metabolism. However, when such reactive species reach concentrations above the upper limit of normal range, they damage cellular components [13, 14, 64]. In this way, therapies that decrease oxidants and/or increase antioxidants are promising in the treatment of various oxidative stress-related inflammatory diseases [37].

Several works showed different therapies as efficient complementary alternatives in disease activity control (Table 1) through antioxidant effects, with improvements observed in many disease activity parameters, such as disease activity score-28 joints (DAS-28), erythrocyte sedimentation rate (ESR), health assessment questionnaire-disability index (HAQ-DI), and visual analog scale (VAS). Among them are N-acetylcysteine, used in other diseases due to its antioxidant effect [37], synbiotic supplementation [62], pomegranate extract [66], coenzyme Q10 [70] and sesamin supplementation [68], rectal insufflation of ozone [35], saline balneotherapy [64], and laser acupuncture [69]. For all adjuvant therapies already described in the literature to date, Table 1 shows the route of administration, dose, duration, and other details from each selected study.

When considering the conventional treatments adequately established for RA, infliximab, an anti-TNF agent, induces beneficial changes in disease activity and also in redox status, evidenced by both the increase in antioxidant defenses and by the decrease in ROS production, through the reduced myeloperoxidase activity and lipid peroxidation [67]. Interestingly, RA patients with coronary artery disease presented increased levels of interleukin-1*β*, protein carbonyl, nitrotyrosine, and MDA than those without the diagnosis of RA. Indeed, in these patients, the inhibition of IL-1 activity by anakinra treatment (single injection 100 mg, SC) reduced the oxidative stress by the reduction of nitrotyrosine, MDA, and protein carbonyl [32]. Similarly, in another study, single injection of anakinra (150 mg SC) decreased MDA, nitrotyrosine, IL-6, and endothelin-1, improving the vascular and left ventricular function in RA patients [75].

Still considering the traditional treatments for RA, methotrexate (MTX), a first-line DMARD in the pharmacological management of RA [30], induced the secretion of IL-10, an anti-inflammatory cytokine, inhibited the production of ^·^NO and increased ROS generation in active RA patients [27]. For these findings, the authors suggest that the decreased ^·^NO levels may contribute to cytokine homeostasis, and the augmented ROS generation may be responsible for MTX apoptotic effect on inflammatory cells, leading to the beneficial action of such DMARD [71].

In addition to conventional pharmacological therapies in the context of RA, other approaches have been described as effective strategies to ameliorate clinical or laboratory parameters in RA patients, either in aspects directly associated to articular compromise or in systemic manifestations related to RA. As an example, in a noninvasive assessment of endothelial function in RA patients, Flammer and colleagues showed that the angiotensin-converting enzyme inhibition by ramipril improved endothelium-dependent vasodilatation, without changes on disease activity or proinflammatory markers [73]. Similarly, Hermann and collaborators also showed an improvement in endothelial function of RA patients after simvastatin treatment during 4 weeks, with this result associated to attenuation of oxidative stress, indicated by a reduction in oxidized low-density lipoprotein (oxLDL) levels and in the oxLDL/LDL ratio [76].

RA patients treated with antioxidant vitamins A, E, and C along with conventional DMARDs for 12 weeks showed decreased levels of MDA and increased concentrations of thiols and reduced glutathione (GSH) and vitamin C concentrations in blood samples. These patients also showed decreased Rheumatoid Arthritis Disease Activity Index (RADAI), suggesting that the antioxidant therapy was efficient in improving both the disease activity and the redox profile in these patients [74]. On the other hand, the increased consumption of antioxidant-rich foods during 3 months did not change the levels of plasma antioxidants and urine MDA in RA individuals. Nevertheless, the plasma levels of retinol presented an inverse correlation with the ESR, DAS-28, and CRP; the vitamin C, a negative relationship with ESR and the HAQ score; the levels of uric acid, in turn, were inversely correlated with the thrombocyte count, pointing to the association between serum uric acid levels and the degree of inflammation in RA patients [77].

Supplementation therapy has been widely described mainly due to its potential antioxidant effect. As a representative of this approach, pomegranate (*Punica granatum L*) is rich in flavonoids, which are potent antioxidants [78]. Its consumption reduced serum oxidative stress in healthy subjects and in atherosclerotic mice [79], as well as in diabetic patients [80]. In RA individuals, pomegranate extract supplementation augmented the concentrations of GPx and reduced the DAS-28 and serum oxidative status [63, 66]. In the same direction, sesamin supplementation decreased serum levels of MDA and increased total antioxidant capacity in RA patients [68]. As another successful complementary therapy, the coenzyme Q10 supplementation, in addition to conventional medications for RA, decreased serum MDA and TNF-*α* levels [70].

Probiotic therapy has also been described in RA individuals. Synbiotic capsule supplements with *Lactobacillus acidophilus*, *Lactobacillus casei*, and *Bifidobacterium bifidum*, for example, improved DAS-28 and VAS pain and increased nitrite, an indirect marker of ^·^NO concentrations, and the GSH in plasma [62]. However, probiotic supplementation containing only *Lactobacillus casei* was not enough to induce significant effect on oxidative stress indices and antioxidant status in such patients [28]. Other different types of nonpharmacological interventions have been proposed as alternative complementary therapies for RA individuals, since clinical and oxidative stress improvements were observed with such approaches. In this regard, Wadley and collaborators [65] demonstrated, in patients with RA, that the aerobic exercise training during 3 months decreased the DAS-28 and the levels of 3-nitrotyrosine, an oxidative stress biomarker. On the other hand, oxidative stress was shown to be increased in response to a single bout of moderate-intensity exercise [65]. In addition, RA patients that performed a 12-session saline balneotherapy in a thermal mineral water pool for 20 min every day increased the nonenzymatic superoxide radical scavenger activity (NSSA) levels, a fact that was accompanied by a significant clinical improvement in terms of patient global assessment [64]. Similarly, complementary treatment with laser acupuncture 3 days/week during 4 weeks diminished oxidative stress and inflammation, ameliorated antioxidant status, reduced ESR values, and improved disease activity (assessed by DAS-28) [69]. Lastly, rectal insufflation of ozone associated with MTX increased the MTX clinical response in RA patients, besides reducing oxidative damage and increasing antioxidant system [35].

## 4. Concluding Remarks

Despite the well-recognized participation of oxidative stress in the pathophysiology of RA, clinical studies devoted to complementary therapies focusing on antioxidant approaches are still scarce. To date, a modest number of trials have shown potential beneficial effects of antioxidant therapies on clinical and biochemical parameters in individuals with RA, shedding light on the perspective of using similar therapies for mitigating disease-related damage, in association with conventional DMARDs. Considering that most of the studies focusing on RA and antioxidant therapies enrolled small numbers of participants with different study designs and distinct methodologies, it becomes difficult to immediately extrapolate their results to RA patients in general, but they represent important research pointing to potential therapeutic interventions to be assessed in larger studies.

Finally, to which extent oxidative stress biomarkers may be useful for early diagnosis of RA and management decisions, for the assessment of disease activity and therapeutic responses, as well as how these potential add-on antioxidant-based treatments will contribute to better disease activity control still hides somewhere. The promising findings from recent research invite us to figure this *conundrum* out, somehow.

## Figures and Tables

**Table 1:** Treatment of rheumatoid arthritis: from conventional approaches to add-on antioxidant therapies.

Authors/year	Enrolled individuals	Therapies/antioxidant/route of administration/dose/duration of treatment	Oxidative effects	General clinical/biochemical effects
Batooei et al., 2018 [37]	RA patients	N-acetylcysteine/oral/600 mg/twice a day for 12 w and conventional medications	Not measured	GH, VAS for the severity of pain, and HAQ scores were improved.

Hirvonen et al., 2017 [36]	RA patients	Whole-body cryotherapy at -110°C, 2 min Whole-body cryotherapy at -60°C, 2 min Local cryotherapy with cold packs or cold air -30°C applied to five swollen joints at a time for 10-30 min All of them were given 3 times/d for 7 d in addition to conventional rehabilitation.	The cold treatment did not increase TRAP after 1 w. However, it induced a short-term increase in the first treatment session at -110°C only.	Not rated

Zamani et al., 2017 [62]	RA patients	Synbiotic capsule supplements^∗^/oral/8 w ^∗^*Lactobacillus acidophilus*, *Lactobacillus casei*, and *Bifidobacterium bifidum* (2 × 109 CFUs/g each) plus 800 mg inulin	Elevation of nitrite (indirect marker of ^·^NO) and GSH in plasma	Reduction in serum hs-CRP levels, improved DAS-28 and VAS pain, and significant reduction in insulin values, HOMA-IR, and HOMA-B

Ghavipour et al., 2017 [66]	RA patients	Pomegranate extract (*Punica granatum L*) contained 40% ellagic acid/oral/2 capsules of 250 mg POMx/once a day for 8 w and conventional medications.	Increased concentrations of GPx; did not change MMP3, CRP, and MDA levels	Reduced DAS-28 and HAQ scores and morning stiffness

Leon Fernandez et al., 2016 [35]	RA patients	Ozone (rectal insufflation) associated with MTX: MTX 12.5 mg, i.m., once/w+ibuprofen 400 mg, orally, one each 8 h+folic acid 5 mg, orally, one/d during 4 d+ozone/20 d (five days/week); 25 mg/l to 40 mg/l of ozone in stepped application and in increasing order was administered. Patients who had been receiving, for at least 3 m before the study, corticosteroid and were under treatment with conventional DMARDs and anti-TNF or other biological agents were excluded.	Reduced anti-CCP levels and oxidative damage, increased antioxidant system; the increased levels of GSH were the only redox marker that correlated with all clinical variables (GSH *vs*. CRP, ESR, DAS-28, and HAQ-DI).	Ozone increased the MTX clinical response.

Karagulle et al., 2016 [64]	RA patients	Saline balneotherapy/2 w:12 balneotherapy sessions in a thermal mineral water pool for 20 min every day except Sunday plus conventional DMARDs/corticoids	Increased NSSA levels	Significant clinical improvement in terms of patient global assessment, physician global assessment, HAQ-DI, DAS-28 based on ESR and swollen joint count, and a trend toward improvement in pain scores

Attia et al., 2016 [69]	RA patients	Laser acupuncture (904 nm,100 mW power output, 1 min irradiation time, beam area of 1 cm^2^, total energy per point 6 J, energy density 6 J/cm^2^, irradiance 0.1 W/cm^2^, frequency 10000 Hz, duty cycle 100%)/3 d/w, with total duration of 4 w plus use of MTX	Decreased oxidative stress, inflammation; improved antioxidant status through increased plasma SOD, GR and CAT activities, and blood GSH; reduced plasma MDA, serum nitrate and nitrite, serum CRP, plasma IL-6 levels; significantly reduced GPx activity	Reduction in ESR and in disease activity (based on DAS-28)

Mateen et al., 2016 [13]	RA patients	Early RA patients were treated with sulfasalazine (1 g/d), deflazacort (6 mg/d), and aceclofenac (100 mg twice/d). Patients with more than 2 years of disease were on sulfasalazine (1 g/d), and NSAIDs were given on irregular basis.	Increased ROS generation, lipid peroxidation, protein oxidation, DNA damage, and impaired enzymatic (SOD, CAT, GR) and nonenzymatic antioxidant (vitamin C and GSH) defense systems; higher MDA content was found in seropositive patients for rheumatoid factor in comparison to seronegative ones. These conditions were worse with the time duration of RA (newly diagnosed, ≤2 years, and between 2 and 5 years)	Increased ESR; patients with 2–5 years of RA duration presented DAS‐28 > 2.4 (meaning active disease)

Abdollahzad et al., 2015 [70]	RA patients	Coenzyme Q10 supplementation capsules^∗^/100 mg/d/2 m ^∗^In addition to their conventional medications (MTX, sulfasalazine, hydroxychloroquine, prednisolone)	Decreased serum MDA and TNF-*α*, without differences in total antioxidant capacity and IL-6 levels	Not rated

Helli et al., 2015 [68]	RA patients	Sesamin supplementation/200 mg/once daily/6 w and conventional pharmacological treatment (MTX, prednisone, sulfasalazine, and hydroxychloroquine)	Decreased serum levels of MDA and increased total antioxidant capacity	Improvement in anthropometric indices, lipid profile, and blood pressure

Vaghef-Mehrabany et al., 2015 [28]	RA patients	Probiotic supplementation/containing 10^8^ CFUs of *Lactobacillus casei* 01, daily capsule/8 w and conventional medications for at least the prior 3 m	No significant effects on oxidative stress indices and antioxidant status	No significant differences for anthropometric parameters, physical activity, anxiety levels, or dietary intakes

Mirtaheri et al., 2015 [81]	RA patients	Alpha-lipoic acid 1200 mg/d for 8 w	Not rated	No differences in serum inflammatory biomarkers (hs-CRP, TNF, and IL-6) and MMP-3 (a marker of joint erosion)

Ikonomidis et al., 2014 [32]	RA patients with coronary artery disease	Single injection of anakinra^∗^/100 mg SC and MTX 7.5 mg once/w, leflunomide 20 mg, and prednisolone 5 mg ^∗^A recombinant IL-1 receptor antagonist	Decreased MDA, nitrotyrosine, and protein carbonyls	Improvement in flow-mediated dilation, coronary flow reserve, arterial compliance, resistance, longitudinal strain, circumferential strain, peak twisting, untwisting velocity, and ejection fraction

Wadley et al., 2014 [65]	RA patients	3 aerobic exercise sessions per week (30–40 min 70% VO_2MAX_) for 3 m in patients with no changes in DMARDs or steroids within the last 3 m	Decreased 3-nitrotyrosine	Decreased DAS-28

Balbir-Gurman et al., 2011 [63]	RA patients	Pomegranate extract (*Punica granatum L*) supplementation/10 ml/day for 12 w in addition to their regular treatment	Reduced serum oxidative status	Reduced DAS-28

Dawczynski et al., 2009 [82]	RA patients	n-3 long-chain PUFA; two groups in a double-blind, placebo-controlled cross-over study; both groups received placebo or verum products consecutively for 3 m with a 2 m washout phase between the two periods. Patients were receiving nonsteroidal anti-inflammatory drugs or corticosteroids.	Did not change biomarkers of oxidative stress	Did not improve disease activity; however, prevented elevated cartilage and bone resorption, favored the diastolic blood pressure, and reduced the lipopolysaccharide-stimulatedCOX-2 expression in plasma

Feijoo et al., 2009 [67]	RA patients	Infliximab/3 mg/kg/administered intravenously at 0, 2, and 6 w All patients were under conventional medications.	Increased GSH, GPx, CAT, SOD, and carbonylated proteins Decreased MPO concentration and lipid peroxidation	Decreased ESR and CRP

Herman et al., 2008 [71]	Active and nonactive RA patients	MTX therapy (7.5-15 mg/kg/w) for at least 6 m before the assessment Folate supplementation was administered 1 to 2 times/w up to 5 mg.	Inhibited the production of ^·^NO and increase in ROS generation in active RA patients	Induced IL-10 secretion in active RA patients

Ikonomidis et al., 2008 [75]	RA patients	Single injection of anakinra (150 mg SC) in patients who had inadequate response to DMARDs and corticosteroids	Decreased MDA and nitrotyrosine levels	Decreased IL-6 and endothelin-1

Flammer et al., 2008 [73]	RA patients	Ramipril (2.5 to 10 mg) for 8 w on top of standard anti-inflammatory therapy	Improved endothelium-dependent vasodilatation No difference in MPO and 8-isoprostane	No difference in DAS-28, blood sedimentation rate, CRP, TNF-*α*, IL-6 and IL-1 Decreased plasma levels of CD40 (a proinflammatory modulator) and diastolic blood pressure

Li et al., 2007 [83]	RA patients	*Ganoderma lucidum* (4 gm) and San Miao San (2.4 gm) daily in addition to their current medications for 24 w	No significant antioxidant effect	Pain score and patient's global score improved significantly only in the *Ganoderma lucidum* group.

Tunez et al., 2007 [40]	RA patients (*n* = 5); ankylosing spondylitis patients (*n* = 5), and psoriatic arthritis patients (*n* = 2)	7 active patients and 5 inactive patients; active patients started therapy with infliximab: RA and psoriatic arthritis (3 mg/kg) and ankylosing spondylitis (5 mg/kg) intravenously at 0, 2, and 6 w. Inactive and control subjects did not receive infliximab therapy. All patients were undergoing treatment with MTX (15 mg/w) and nonsteroidal anti-inflammatory agents. RA and psoriatic arthritis patients were also receiving 10 mg/d of prednisone.	Infliximab protected against oxidative stress triggered in patients with active disease (decreased protein carbonyls and increased GSH, GSH-peroxidase, CAT, and SOD).	BASDAI and DAS-28 were decreased in ankylosing spondylitis and in RA active patients, respectively.

Herrera et al., 2006 [84]	Patients who were prescribed by their private physicians mycophenolate mofetil for the treatment of psoriasis (*n* = 3) or RA (*n* = 5) and had a grade I essential hypertension and normal renal function	Mycophenolate mofetil therapy, during 3 m; initial dose was 1 g/d and increased over 1 w to 1.5 to 2.0 g/d administered in two divided doses. Four RA patients received throughout the study prednisone 5 mg/d and one received chloroquine 2 tabs/d and captopril 25 mg twice daily.	Plasma and urinary excretion of MDA did not decrease significantly.	Reduction in systolic, diastolic, and mean blood pressure and urinary excretion of TNF-*α*; CRP levels and urinary IL-6 and MCP-1 excretion did not show consistent changes.

Hermann et al., 2005 [76]	RA patients with normal cholesterol levels	Simvastatin 40 mg/day for 4 w^∗^ ^∗^Antirheumatic drug therapy was unchanged for 3 m before inclusion in the study and remained stable throughout.	Improved endothelial function and decreased oxidative stress indicated by a reduction of oxLDL levels and the oxLDL/LDL ratio	Reduced total cholesterol, LDL cholesterol, apolipoprotein B, and aspartate aminotransferase

Jaswal et al., 2003 [74]	RA patients	Antioxidant vitamins A, E, and C along with the conventional drugs for 12 w	Increase in thiols, GSH, and vitamin C Decreased MDA	Decreased RADAI

Hagfors et al., 2003 [77]	RA patients	Increased consumption of antioxidant-rich foods during 3 m and conventional medications; Modified Cretan Mediterranean Diet: fruits, vegetables, pulses, cereals, fish with a high content of *ω*-3 fatty acids, nuts and seeds with a high content of *α*-linolenic acid, teas, olive oil, canola oil, and the liquid and half-fat margarines based on canola oil. The Mediterranean Diet group was advised to replace high-fat dairy product for low-fat products.	No change in the levels of plasma antioxidants and urine MDA	Inverse correlation between retinol and ESR, DAS-28, and CRP; negative relationship between vitamin C and ESR and vitamin C and the HAQ score; uric acid negatively correlated with the thrombocyte count.

Anti-CCP: anticyclic citrullinated peptide antibody; BASDAI = Bath Ankylosing Spondylitis Disease Activity Index; CAT = catalase; CFUs: colony forming units; COX-2 = cyclo-oxygenase 2; CRP = C-reactive protein; d = day; DAS-28 = disease activity score-28 joints; DMARDs = disease-modifying antirheumatic drugs; ESR = erythrocyte sedimentation rate; GH = global health; gm = gram; GPx = glutathione peroxidase; GR = glutathione reductase; GSH = reduced glutathione; HAQ = health assessment questionnaire; HAQ-DI = health assessment questionnaire-disability index; HOMA-IR = homoeostasis model of assessment-estimated insulin resistance; HOMA-B = homoeostatic model assessment-*β*-cell function; hs-CRP = high-sensitivity C-reactive protein; i.m. = intramuscular; IL-1 = interleukin 1; IL-6 = interleukin 6; IL-10 = interleukin 10; LDL = low-density lipoprotein; m = months; MCP-1 = monocyte-chemoattractant protein-1; MDA = malondialdehyde; MMP3 = matrix metalloproteinase 3; MPO = myeloperoxidase; MTX = methotrexate; ^·^NO = nitric oxide; NSSA = nonenzymatic superoxide radical scavenger activity; oxLDL = oxidized low-density lipoprotein; POMx = pomegranate extract; PUFA: polyunsaturated fatty acids; RA = rheumatoid arthritis; RADAI = Rheumatoid Arthritis Disease Activity Index; ROS = reactive oxygen species; SC = subcutaneous; SOD = superoxide dismutase; TNF = tumor necrosis factor; TRAP = total radical-trapping antioxidant parameter; VAS = visual analog scale; w = weeks.
